# Overview of the Assessment of Endothelial Function in Humans

**DOI:** 10.3389/fmed.2020.542567

**Published:** 2020-10-07

**Authors:** Po Ying Chia, Andrew Teo, Tsin Wen Yeo

**Affiliations:** ^1^National Centre for Infectious Diseases, Singapore, Singapore; ^2^Lee Kong Chian School of Medicine, Nanyang Technological University, Singapore, Singapore; ^3^Department of Infectious Diseases, Tan Tock Seng Hospital, Singapore, Singapore; ^4^Department of Medicine and Radiology and Doherty Institute, University of Melbourne, Victoria, VIC, Australia

**Keywords:** endothelial dysfunction, diagnostic markers, biomarkers, microvascular, macrovascular, vascular tone, anticoagulation, permeability

## Abstract

The endothelium is recognized to play an important role in various physiological functions including vascular tone, permeability, anticoagulation, and angiogenesis. Endothelial dysfunction is increasingly recognized to contribute to pathophysiology of many disease states, and depending on the disease stimuli, mechanisms underlying the endothelial dysfunction may be markedly different. As such, numerous techniques to measure different aspects of endothelial dysfunction have been developed and refined as available technology improves. Current available reviews on quantifying endothelial dysfunction generally concentrate on a single aspect of endothelial function, although diseases may affect more than one aspect of endothelial function. Here, we aim to provide an overview on the techniques available for the assessment of the different aspects of endothelial function in humans, human tissues or cells, namely vascular tone modulation, permeability, anticoagulation and fibrinolysis, and the use of endothelial biomarkers as predictors of outcomes.

## Introduction

The endothelium consists of a single cell layer of endothelial cells, lining the vascular and lymphatic systems, and covers a surface area of more than 1,000 m^2^ (1). The apical surface of the endothelium is covered by a layer called the endothelial glycocalyx (2), which consists of a mosaic of glycoproteins and proteoglycans, and glycosaminoglycan chains. The endothelial glycocalyx, together with secreted proteoglycans and other adsorbed plasma proteins including albumin, forms the endothelial surface layer (3).

Endothelial cells from different vascular sites and organs have demonstrated variations in their appearance and surface constituents (4–6). Likewise, the corresponding endothelial glycocalyx have also been shown to be different in different organ beds (7). This phenotypic heterogeneity allows the endothelium to serve an array of functions tailored for different sites.

In the 1950s, the endothelium was believed to be merely a “sheet of nucleated cellophane” (8), however the importance of the endothelium in haemostasis and arterial smooth muscle relaxation, and thus vascular tone, was subsequently recognized (9, 10). The term “endothelial dysfunction” was then coined following the observation of diminished pulmonary exchange after intratracheal administration of bleomycin in rabbits (11). Currently, the endothelium is known to play an important role in the modulation of vascular tone, dynamic permeability, thrombogenicity, inflammation, and angiogenesis (12–14). These functions are achieved via receptors and release of a diverse family of compounds such as autacoids like prostacyclin, endothelin-1, and angiotensin II (13).

Given the diversity in function and heterogeneity of the endothelium, measurement of endothelial function is challenging. Endothelial function in certain aspects are studied in greater detail compared to others due to earlier understanding of certain physiology and the availability of technology. For example, endothelial function in terms of angiogenesis is not well-studied *in vivo* in humans, although there are various assays and guidelines on *in vitro* interpretation (15, 16), while endothelial function in terms of vascular tone modulation has been much more widely studied (17).

In this review of endothelial function measurement, we will be concentrating on areas where techniques to measure endothelial function in humans or human cells and tissues are well-established. The sections are categorized into: (1) vascular tone modulation and tissue perfusion, (2) dynamic permeability, (3) anticoagulation and fibrinolysis, and (4) endothelial function biomarkers. Each section is then further categorized into broad physiological phenomena utilized (if relevant), then subcategorized into *in vivo, ex vivo*, or *in vitro* techniques and then the method itself. We will summarize various techniques used to quantify endothelium function, the underlying evidence, and clinical associations reported. Many of the existing techniques measure surrogate endpoints, as we are unable to directly measure endothelial function. Techniques detailed in the same section should thus, not be treated as equivalent to each other (18, 19).

We categorized the methods according to the endothelial function being assessed and underlying physiological principle utilized, for ease of reference, although certain methods may be able to assess more than one function of the endothelium. As this review is meant to be an overview of methods available to measure endothelial function, the physiological basis behind the measurements and limitations will only be briefly mentioned (Table 1).

**Table 1:** Summary of techniques in the measurement of endothelial function.

**Technique**	**Invasiveness**	**Experiment type**	**Areas or parameters assessed**	**Specific limitations if any**	**Examples of clinical disease**	**Commercial devices available**
**VASCULAR TONE**						
Intra-arterial or intra-venous infusion of vasoactive substances	Invasive	*In vivo*	Artery, venous, and microvasculature of study area	- Depending on vasoactive substance, and site of infusion, response measured may not be due to endothelium alone- Time consuming	Cardiovascular disease, smoking, hyperglycaemia, (20, 21)	Yes
**POST-OCCLUSIVE REACTIVE HYPERAEMIA**						
- Flow mediated dilation	Non-invasive	*In vivo*	Artery and microvasculature of study area (If using ultrasound: limited to forearm)	- If using ultrasound, high degree of operator skill required.- If using magnetic resonance imaging, it is expensive.- Strict protocol to follow.	Cardiovascular disease, hypercholesterolemia (22–24)	Yes
- Enclosed Zone Flow Mediated Dilation	Non-invasive	*In vivo*	Arteries of limbs	- Not as well-studied as flow mediated dilation	Cardiovascular disease (25)	Yes
- Laser based techniques	Non-invasive	*In vivo*	Skin microvasculature	- Laser required- Strict standardization of study site required.	Cardiovascular disease, diabetes (26–28)	Yes
- Digital reactive hyperemia index in peripheral arterial tonometry	Non-invasive	*In vivo*	Microvasculature of fingertips	- Disposable finger plethysmographic probes required, which can be costly	Cardiovascular diseases, hypertension, diabetes, smoking, infection, and autoimmune disease (29–33)	Yes
- Venous occlusion plethysmography	Non-invasive	*In vivo*	Venous system of limbs	- Strict protocol to follow.	Cardiovascular disease, vascular claudication, diabetes (34–36)	Yes
**ARTERIAL STIFFNESS**						
- Arterial compliance, pulse wave analysis	Non-invasive	*In-vivo*	Arteries	- Strict protocol to follow.	Cardiovascular disease, obesity, hypertension, smoking, diabetes (37–39)	Yes
- Atomic force microscopy	Invasive	*Ex-vivo*	Endothelial glycocalyx	N. A.	Cardiovascular disease, diabetes, infections, inflammatory disease, cancer (40–42)	Yes
- Myograph	Invasive	*Ex-vivo*	Arteries	- Opportunistic sampling	Cardiovascular disease, pre-eclampsia (43–45)	Yes
- Endothelial cell culture	N.A.	*In vitro*	- Cellular nitric oxide release - Flow-induced nitric oxide release	- Important *in vivo* characteristics may be lost, however this may be modulated with microfluidic models	Cardiovascular disease, diabetes, hypertension, pulmonary arterial hypertension (46, 47)	Yes
- Intra-vital microscopy e.g., OPS, SDF or IDF	Non-invasive	*In-vivo*	Microvasculature, endothelial glycocalyx	N. A.	Cardiovascular, infection, pulmonary arterial hypertension (48–50)	Yes
- Microdialysis	Invasive	*In-vivo*	Microvasculature	N. A.	Infection (51)	Yes
**DYNAMIC PERMEABILITY**						
- Venous occlusion plethysmography	Non-invasive	*In vivo*	Capillaries	- Strict protocol to follow.		Yes
- Transendothelial electrical resistance	N.A.	*Ex vivo—In vitro*	Ionic conductance of paracellular pathway	- Cultured endothelial monolayer in the absence of flow shear stress have impaired barrier function, however this may be modulated with microfluidic models	Neurological disorders Infection, inflammatory disease (52–55)	Yes
- Transendothelial flux of labeled macromolecules	N.A.	*Ex vivo—In vitro*	Paracellular water flow and pore size of tight junction	- Cultured endothelial monolayer in the absence of flow shear stress have impaired barrier function, however this may be modulated with microfluidic models	Diabetes, pre-eclampsia (56–58)	Yes
**ANTI-COAGULATION AND FIBRINOLYSIS**						
- Tissue plasminogen activator release	Invasive	*In vivo*	tPA release from forearm vasculature	N.A.	Cardiovascular disease, hypercholesterolemia, hypertension, smoking (59, 60)	Yes—Assay to measure tissue plasminogen activator
- Functional assay to neutralize FXa and thrombin	Invasive	*Ex vivo—In vitro*	Ability to neutralize FXa and thrombin	N.A.	Autoimmune disease (61, 62)	Yes—Assay to measure FXa and thrombin
**OTHERS**						
Endothelial function biomarkers	Non-invasive to Invasive	*Ex vivo*	Systemic vasculature including endothelial glycocalyx	- Kinetics may not be well-known	Cardiovascular, infection, cancer, autoimmune, pre-eclampsia, and eclampsia (63–67)	Various ELISA kits may be available

## Vascular Tone Modulation and Tissue Perfusion

In healthy vessels, the endothelium regulates vascular tone locally via autacoids including prostacyclin and nitric oxide (NO), which have been broadly categorized into endothelium-derived relaxing, hyperpolarizing and contracting factors (68). NO is a well-known and well-studied molecule that is predominantly characterized as an endothelium-derived relaxing factor (69). NO activates soluble guanylyl cyclase in vascular smooth muscle cells, resulting in cyclic guanosine monophosphate production and triggering relaxation (70). Stimulators for the release of endothelium-derived relaxing factors reported include serotonin, thrombin, adenosine, bradykinin, and insulin (71). The endothelium can also release endothelium-derived contracting factors such as vasoconstrictor prostanoids and endothelin-1, which stimulates the vascular smooth muscle cells to contract (71). Endothelium dependent dilation (EDD) is thus dependent on a multitude of factors, ranging from NO production, prostanoids, endothelin-1, and other endothelium derived hyperpolarizing factors (71).

Endothelial dysfunction in terms of vascular tone is categorized as any phenotype leaning toward a vasoconstrictive profile (72, 73) and may arise from reduction in production of relaxing mediators or altered endothelial response to relaxing mediators. Blunting of EDD is observed in patients who have traditional cardiovascular risk factors such as smoking (74) and is a predictor of future cardiovascular disease (75, 76). Overall vascular tone modulation or vasodilatory response to ischemic stimulation is a surrogate measure of endothelial function.

EDD is commonly used to determine endothelium dysfunction in vascular tone modulation. Besides measuring EDD response to direct chemical stimulus, physiological responses namely reactive hyperaemia can also be utilized. Post-occlusive reactive hyperaemia (PORH) describes the phenomenon where blood flow increases after a period of arterial avascularisation or ischaemia (77), and is in part, due to EDD depending on the vascular site analyzed. Characteristics of blood flow, including peak blood flow, is measured pre- and post-ischaemic challenge through inflation of a cuff at rest to temporarily stop blood flow or via exercise (17, 78, 79). Sites of measurements may include arteries, veins and capillaries using different methods as follows (17, 78, 79).

Other measures of vascular tone modulation besides measuring EDD include measuring arterial stiffness or compliance and analyzing the pulse wave characteristics (80). Arterial compliance can be quantified by the difference between change in vessel diameter in systole and diastole or pulse wave velocity. Potential sites of measurement include the brachial artery, carotid artery or aorta. Characteristics of the pulse wave including velocity can be quantified by pulse contour analysis. Pulse wave velocity is obtained by measuring the speed of the arterial pressure wave propagation and is inversely related to arterial wall stiffness.

### Endothelium Dependent Dilation and Others

#### *In vivo* Techniques

##### Intra-arterial or intra-venous infusion

In the arteries, a few methods are established to measure EDD. One of them is intra-brachial infusion of acetylcholine, which evokes EDD measurable by ultrasound (81). Acetylcholine is the most frequently prescribed endothelium-dependent vasodilators in experimental models and human research studies. Acetylcholine binds to muscarine receptors and activates endothelial NO synthase, stimulating the production of NO from L-arginine. Other NO agonist such as methacholine, and bradykinin can also be used (82). Intra-arterial infusion of other vasoactive substance can also be used to measure endothelial reaction, and thus dysfunction if reaction is impaired.

Besides intra-arterial infusion, intravenous infusion of vasoactive compounds can also be performed to assess endothelial function (83). Dorsal hand vein technique can be used to determine venous tone and was first described in 1981 (84). A needle is inserted into a vein on the hand dorsum and a linear transformer is connected to the needle to measure vein diameter after pre-treatment with vasoactive compounds. Depending on the vasoactive substance infused, underlying physiological reactions may be different and may not be purely due to EDD. While intra-arterial and intra-venous infusion methods are fairly safe, they are still invasive, and more time-consuming than other methods available (82).

##### Flow mediated dilation

Flow mediated dilation (FMD) is a common method used to measure vascular endothelial function of the brachial artery and utilizes imaging, commonly ultrasound, to measure the arterial dilation during PORH. PORH can be achieved thorough inflating a pneumatic pressure cuff around the arm to suprasystolic pressure for 5 min. Reactive hyperaemia is then generated by rapidly releasing the pressure and in the brachial artery, this phenomenon is due to NO-mediated EDD. The brachial artery is thus a favored site for measuring FMD as NO is the sole mediator of FMD in the brachial artery, and provides a more accurate surrogate measure of endothelial NO production (85). Impaired dilation suggests impaired release of endogenous vasodilator in response to ischemia and thus endothelial dysfunction.

Maximal artery diameter is recorded and the FMD%, an index, is then calculated from the pre- and post-occlusion maximal artery diameter (22, 86). Hyperaemic blood flow velocity can also be calculated in the same setting (87, 88), however scores obtained from measuring blood flow velocity e.g., pulsatility index or resistance index are not as well-studied as compared to FMD for measurement of endothelial function (89, 90). Arterial stiffness can also be calculated and is elaborated in the corresponding section below.

The use of ultrasound for FMD limits its use to easily visualized arteries, requires a high frequency transducer, and skilled ultrasonologist for accurate measurements. Otherwise, large errors in flow estimation can occur (91). A standardized protocol on subject preparation and assessment of FMD is also required to ensure reproducibility (86, 92, 93). Other methods to measure FMD includes magnetic resonance imaging (MRI), and this has been validated against ultrasound (94). Use of MRI allows PORH of deeper arteries to be visualized and limits inter-observer differences but requires the use of more expensive equipment and highly skilled personnel, which may not be practical in low resource settings.

New techniques including enclosed zone FMD (ezFMD) have been developed to circumvent some of the abovementioned limitations. In ezFMD, oscillometry is used to measure changes in oscillation amplitude of intra-arterial pressure changes and thus variation in vascular volume (25). Change in vascular dilatation in response to ischaemia can potentially quantify lower-limb vascular endothelial function. As oscillometry is used, ezFMD readings can be obtained using an air cuff with minimal technical expertise and invasiveness.

On a side note, low-flow-mediated-constriction (L-FMC) can be measured in the same setting as FMD and provides additional information beyond FMD on endothelial dysfunction (95, 96). L-FMC involves measuring the diameter and flow in the last segment of arterial avascularization and thus vascular reactivity. The measured L-FMC is likely to be due to a combination effect of both endothelial derived relaxing and contracting factors and not EDD alone. L-FMC is expressed as the percentage of the decrease in diameter during arterial avascularization with resting diameter (95, 96).

##### Reactive hyperaemia index—peripheral arterial tonometry

PORH has also been studied in microvasculature and is measured as reactive hyperaemia index (RHI) and was first developed in 2003 (97). EDD is measured by the digital pulse waveform, also known as peripheral arterial tone. Disposable modified plethysmographic probes on the index fingers measure the digital pulse waveform pre and post-occlusion of the brachial artery (98). Pre-and-post-occlusion values are then used to calculate the RHI. A linear relationship between RHI and FMD was demonstrated and further studies and a meta-analysis has demonstrated association with cardiovascular events (99).

##### Venous occlusion plethysmography

The use of venous occlusion plethysmography to measure blood flow was first described in the 1900s (100). Venous return from the limb or area of study is interrupted via inflation of a cuff to below diastolic pressure, allowing for arterial inflow and venous emptying. Blood flow is then measured by change of volume over a fixed duration. The maximal blood flow is influenced by vascular resistance, and thus vascular tone. Venous occlusion plethysmography therefore can be used to indirectly measure local vascular tone in response to an ischaemic challenge. Change in limb volume can be measured via air filled, water filled, mercury plethysmography or impedance plethysmography (101–104). Additional modifications to this technique include combination with intra-arterial drug administration and the use of automation. While the results appeared repeatable and reproducible, variation in factors such as cuff inflation time may significantly affect blood flow recorded (105).

##### Laser Doppler

Laser Doppler flowmetry (LDF) is another non-invasive technique used to measure EDD of skin microvasculature post stimuli (106). The laser light penetrates skin and soft tissue and is then partially backscattered by red blood cells. Based on the Fizeau Doppler principle, blood flow velocity is then derived from the frequency of the backscattered light (107). The first developed laser Doppler technique was flowmetry, otherwise known as Doppler perfusion monitoring, where skin microvasculature was assessed over a small volume of maximum 1 mm^3^. However, due to regional heterogeneity of skin microvasculature and the small volume assessed, the reproducibility is poor (108). With improvement in technologies, laser Doppler imaging (LDI) or laser Doppler perfusion imaging (LDPI) has allowed for a larger volume of interest to be imaged and quantified (106, 109), this may result in improvement in reproducibility. Laser speckle contrast imaging (LSCI) utilizes a widespread laser, forgoing a grid scanning process required in the previous techniques, allowing faster imaging (110, 111). A range of stimuli can be used to stimulate the endothelium of the skin microvasculature as well, including PORH, local thermal hyperaemia, iontophoresis or transdermal application of acetylcholine, and microdialysis to introduce drugs of interest (109, 112, 113). Laser-based techniques have also been used in clinical use where assessment of microcirculation is warranted, such as in wound and burn assessments (114).

#### *Ex vivo* and *in vitro* Techniques

##### Endothelial cell culture

Culture of human endothelial cells was described in 1960s (115) and has been used for high spatiotemporal imaging of intracellular processes and analyses of signaling molecules without background noise from other cell types (116). Direct measurement of mediators of specific stimuli on mediators of vascular tone, specifically NO release or reactive oxygen species can be performed via endothelial cell culture using different assays (117). An example would be the commonly used 4,5-diaminofluorescein (DAF-2) and cell permeable DAF-2 diacetate assay (117, 118), which quantifies real-time NO release from human endothelial cells.

### Arterial Stiffness

#### *In vivo* Techniques

##### Arterial compliance and pulse wave analysis

The use of blood vessel imaging with ultrasound or magnetic resonance in measuring FMD allows the measurement of arterial compliance with sonographic stiffness indices such as augmentation index (94, 119). Pulse wave velocity also allows measurement of arterial stiffness, with carotid-femoral pulse wave velocity being the gold standard for large artery stiffness (120). One-point pulse wave velocity and oscillometric methods have also been developed to measure arterial compliance easily (121, 122). Arterial stiffness is generally increased in persons with cardiovascular risk factors and heart failure, however it is not uniform across all arteries (37). As routine clinical measurement is not practical, pulse wave velocity measurement is not yet recommended for routine practice (123). Improvements to pulse wave velocity is possible with the use of angiotensin converting enzyme inhibitors and angiotensin receptor blockers (124), however clinical implications of this improvement has not been well-demonstrated (125).

#### *Ex vivo* Techniques

##### Myography

*Ex vivo* methods such as myography has been developed to measure arterial stiffness in association to endothelial dysfunction. Wire myography in 1970s was developed to study contractile responses of small resistance arteries (126) and since then other modifications such as isobaric and isotonic conditions have been utilized to optimize experimental studies (127, 128). Human mesenteric microarteries mounted in myographs have demonstrated an association between age and presence of cardiovascular risk factors with EDD (129). However, in view of the invasiveness, sampling is often opportunistic in human studies or from subcutaneous fat (130, 131).

##### Atomic force microscopy

Atomic force microscopy (AFM) nano-indentation technique developed in 1986 (132) has also been utilized to measure the thickness and stiffness of the endothelial glycocalyx (133) which can be a prognostic marker for endothelial dysfunction. A triangular cantilever with a spherical tip periodically indents the endothelium and a laser beam is then used to measure the cantilever flection. The thickness of the endothelial glycocalyx is then calculated from the force on the cantilever, piezo displacement and deflection sensitivity. AFM can be used in *in vitro* or *ex vivo* experiments of blood vessel preparations (133, 134).

### Intra-Vital Microscopy

Direct visualization of the microvasculature using hand-held intravital microscopes at the bedside has also been used in recent years (135). Microvasculature function includes endothelial function and leukocyte-endothelium interaction (136). Stagnant flow, red blood cell flow velocity can be directly observed and glycocalyx thickness indirectly observed using this method. Orthogonal polarization spectral (OPS) imaging is considered the first generation of hand-held intravital microscopes and was developed in 1990s (137). However, due to motion-induced image blurring in OPS imaging, sidestream dark field (SDF) imaging was developed with improvements in capillary contrast and quality (138). Incident dark field (IDF) imaging was then introduced subsequently in 2015 with improved resolution and visualization of more capillaries (139). An excellent review on the technical aspects has been written by Massey and Shapiro (140). Intravital microscopy is predominantly performed in the sublingual area and involves gently holding a hand-held pen-like-camera onto the study area. Intra-vital microscopy has correlated the thickness of the endothelial glycocalyx with microvascular perfusion (141), and use in sepsis have demonstrated thinning of the endothelial glycocalyx to be independently associated with worse clinical outcomes (142–144).

### Microdialysis

Microdialysis can also be used for real time measurement of reactive oxygen species (ROS) in the extracellular environment as a function of endothelium. The technique involves inserting porous probes into target tissue and perfusing it with physiological saline, allowing real-time and continuous passive diffusion of molecules from extracellular fluid into a dialysis bag. The use of a dialysis bag to collect small amounts of samples from interstitial tissue was first described in 1966 in animal brains (145), and has since been used in a variety of tissues to explore different molecular pathways (146). The collected microdialysate can then be assayed with enzyme linked immunosorbent assay (ELISA) or liquid chromatography/tandem mass spectrometry for various molecules (LC/MS) (147). In the setting of quantifying endothelial dysfunction, ROS levels in interstitial tissues can be an indirect measure of tissue perfusion, and studies have associated microvascular endothelial dysfunction with elevated ROS levels (148).

## Dynamic Permeability

Endothelial cells are connected to each other via intercellular junctions including tight junctions and adherens junctions (149). These intercellular junctions regulate passive diffusion and allow the endothelial layer to form a selectively permeable layer to maintain homeostasis. Transport of molecules smaller than 3 nm occurs paracellularly while transport of macromolecules such as albumin occurs transcellularly in physiological conditions (150). The endothelium of different organ beds has different barrier functions and techniques for measuring endothelial dysfunction are not universally applicable.

### *In vivo* Techniques

The use of venous occlusion plethysmography to quantify vascular permeability was picked up in the 1960s (151), as there were initial technological difficulties in measuring small blood-tissue fluid shifts due to changes in capillary permeability when the predominant volume changes during venous occlusion occurred intravascularly (152). Nevertheless, venous occlusion plethysmography has subsequently been used to measure the permeability of capillaries in the form of the capillary filtration coefficient (CFC) (151, 153, 154). CFC consists of two components; one is the hydraulic conductance of the blood vessel and second is the surface area. Compared to measurement of PORH, the use of venous occlusion plethysmography to measure CFC involves occlusion via multiple steps of small increases in cuff pressure rather than one single step. The small increases in cuff pressure diminishes the effect of PORH and allows the increase in limb volume with each increase in cuff pressure to be due to filling of capacitance blood vessels and fluid efflux from the microvasculature. CFC is then obtained via least squares fitting of the volume response after filling of capacitance blood vessels is completed (151). Although CFC has been described in various disease processes, care must be exercised in documentation site of measurement and cuff pressure increase increment to ensure reproducibility (155, 156).

### *In vitro* Techniques

#### Transendothelial Electrical Resistance

*In vitro* experiment using cultured endothelial cell monolayers allows for transendothelial electrical resistance (TEER) to be measured as a surrogate measure of the barrier function (52). TEER measures ionic resistance which consists of resistance associated with paracellular ion transport and ion transport across apical and luminal cell membranes. TEER can be measured via Ohm's law method which involves endothelial cell monolayers being cultured on a semipermeable filter in a two-compartment chamber, with an upper compartment and a bottom compartment. Electrodes are then inserted into the upper and lower compartments and are separated by the cultured endothelial cell monolayer. A direct or alternate current is then applied across the electrodes and the resulting current measured. TEER is then calculated based on Ohm's law.

Impedance spectroscopy is another method used to measure TEER. A small alternate current is applied across the electrodes and the amplitude and phase response of the resulting current is then measured. TEER is then calculated using the characteristics of the resulting current and phase shift. An excellent review on TEER has been published by Srinivasan et al. (52). TEER pre- and post-stimulus is recorded and results are reported as a percentage of baseline (52). Although TEER has been used *in vivo* animal studies (157), this technique is currently too invasive in humans, and is thus characterized under *in vitro* techniques.

#### Transendothelial Permeability

Transendothelial permeability of labeled large molecules, ranging from carbon-14 labeled sucrose to fluorescein isothiocyanate (FITC) labeled albumin or dextran, across endothelial monolayers is another commonly used method to measure permeability (158). Macromolecule diffusion across endothelial monolayers can also be quantified using enzymatic markers such as horseradish peroxidase (159, 160) or visualized using confocal microscopy or electron microscopy (161, 162).

Barrier function in cultured endothelial monolayers alone however are not fully representative of *in vivo* conditions (163). There is variation in the reported thickness of the endothelial glycocalyx in cultured endothelial cells and *ex vivo* tissue (164), although the differences may be contributed by different in techniques (165, 166). Flow shear stress has also been demonstrated to enhance barrier function of the endothelium (167, 168) and formation and maintenance of healthy endothelial glycocalyx is dependent on the presence of flow (169). Technological advancements have since allowed optimization of the microenvironment of endothelial cell cultures to better replicate *in vivo* conditions including the establishment of flow shear stress. These methods have been further improved to resemble physiological conditions using tissue-on-a-chip model and can be generally classified as two-dimensional microfluidic models, hybrid microfluidic models, three-dimensional templated models, or self-organization models (170, 171). Despite these advances, models matching physiological microenvironment are still unavailable (172).

### *Ex vivo* Techniques

The measurement of protein or albumin loss in the urine is actively used in clinical evaluation of endothelial dysfunction in terms of permeability. In other end organs, such as the lung, the amount of total protein concentrations or other labeled proteins in the bronchioalveolar lavage fluid can also be measured as surrogate markers to endothelial permeability (173). The analysis of bronchioalveolar lavage fluid however requires bronchoscopy to be performed and is considered invasive.

Human vascular endothelial cells can also be extracted via J-wires, pulmonary artery catheter balloons and other organ endothelial cells via surgery such as during tumor resection and cultured to evaluate site-specific barrier function with different stimuli (174–177). For example, barrier dysfunction was demonstrated via leakage of Evans blue dye-albumin across a monolayer of cultured human lung microvascular endothelial cells but not human umbilical vein endothelial cells under experimental sepsis conditions (176). Protein expression on the surface of these endothelial cells and chemokines production can also be analyzed to evaluate endothelial dysfunction (178). The breakdown of endothelial glycocalyx also hampers the dynamic permeability of the endothelium and will be expounded upon in the body fluid biomarkers section below.

## Anticoagulation and Fibrinolysis

Tissue plasminogen activator (tPA), a key enzyme in the fibrinolytic pathway, is released by the endothelium and protects against development of atheroma, activation of platelets and formation of thrombus (179). Techniques for quantifying the *in vivo* release of tPA from the endothelium in response to stimuli have been developed (180) and can function as a measure of anti-thrombotic function. The measurement of tPA release is usually performed in the same setting with venous occlusion plethysmography for forearm blood flow (181, 182). Drugs such as substance P are infused via brachial artery cannulation and the release of tPA in response is then measured in the venous blood sampled from the subcutaneous veins in the antecubital fossa (180). In persons with traditional cardiovascular risk factors such as older age coupled with sedentary lifestyle, smoking and coronary artery disease, the release of tPA is reduced (20, 59, 183).

Functional analysis of anticoagulant activity in terms of ability to inactivate Factor Xa (FXa) and thrombin *in vitro* on endothelial tissues or cultured cells can also be performed (61). The tissue or cells are incubated with a fixed volume of antithrombin (AT) for 5 min, allowing the activation of AT through binding of surface-exposed heparan sulfate. A fixed amount of FXa or thrombin is then added and incubated for 2 min to allow activated AT to bind and inactivate FXa or thrombin. The supernatant is then analyzed for free FXa or thrombin. The amount of free factor Xa or thrombin thus indirectly measures the anticoagulation function of the endothelium.

## Endothelial Function Biomarkers

Several biomarkers associated with endothelial function with clinical correlates have been identified, for example, studies have correlated levels of angiopoietins, selectins, and growth factors with severity of sepsis (63, 184, 185). Majority of the biomarkers can be measured with ELISAs or LC/MS. However, the kinetic data of these biomarkers for endothelial injury are not well-known, and as such studies of these biomarkers may have superficially contradictory results and reproducibility may depend on severity and duration of illness during measurement (63).

Angiopoietins (Ang) 1 and 2 belong to the family of growth factors and are involved in angiogenesis, as well as other functions including vascular permeability (184). Levels of Ang 1 and 2 in plasma both correlate with mortality and disease severity in sepsis (64, 186, 187). Angiopoietin like proteins (ANGPTL) are a family of proteins, of which eight have been described (188). Like angiopoietins, ANGPTL regulates angiogenesis and some may exhibit other functions and may be potential biomarkers for endothelial function. For example, ANGPTL 2 is a circulating glycoprotein and a proinflammatory mediator (188). In a study involving Japanese adults, ANGPTL 2 was found to be independently associated with risk of diabetes (189), and is possibly a key molecule linking obesity with insulin resistance and subsequent occurrence of diabetes. ANGPTL 6 might also play a role in endothelial function as higher levels were associated with pregnancy-induced hypertension (190).

Selectins expressed on activated endothelial cells may also be shed and measured in circulation, reflecting endothelial injury, and thus endothelial function (191). Selectins are key adhesion molecules and modulate leukocyte movement (192). Similar to selectins, intercellular adhesion molecule-1 (ICAM-1), vascular cell adhesion molecule-1 (VCAM-1), and platelet endothelial cell adhesion molecule-1 (PECAM-1) also modulate leukocyte adhesion and movement (63, 193). Studies have correlated increased levels of selectins, ICAM-1 and/or VCAM-1 with sepsis although there are contradicting studies (63).

Growth factors such as vascular endothelial growth factor (VEGF) and soluble VEGF receptor-1 (VEGFR-1), also known as Flt-1, can also be quantified in circulation. VEGF and its receptors stimulates endothelial cell migration, hyperpermeability, and angiogenesis (194) and levels of VEGF and Flt-1 are both elevated in diseases with endothelial dysfunction such as sepsis and pre-eclampsia (195–197). VEGFR-2, also known as fetal liver kinase-1 (Flk-1) or kinase domain-containing receptor can also be measured in the serum, and may be a promising biomarker for certain cancers (198, 199). Current available angiogenesis assays are mainly used to assess anti-angiogenic or pro-angiogenic factors and not endothelial function (16). Thus, measurement of circulating biomarkers may be the closest method to measuring endothelial function in terms of angiogenesis.

Platelet derived growth factor (PDGF) also has multiple functions (200) and low levels can result in increased permeability (201). There are other potential biomarkers such as plasma soluble endoglin, a receptor of transforming growth factor-beta, which requires further evaluation. Levels of plasma soluble endoglin directly correlates with complicated diabetes and hypertension with end-organ damage, higher 10 year cardiovascular risk scores (202), pre-eclampsia (65), and yet an inverse correlation with severity of coronary artery disease (203).

Quantification of breakdown products of the endothelial glycocalyx layer has also gained interest in recent years. Breakdown products of the endothelial glycocalyx layer has been shown to correlate with endothelial dysfunction in metabolic diseases (204, 205) including inborn errors of metabolism (206), infections such as malaria, dengue and sepsis, and acute respiratory distress syndrome (207–210). Measurable endothelial glycocalyx breakdown products in the plasma or serum include syndecan-1, chondroitin sulfate, dermatan sulfate, serum hyaluronic acid, and heparan sulfate (208, 211).

Syndecan-1 was the first proteoglycan characterized (212), and is a cell surface heparan sulfate proteoglycan that is released in endothelial glycocalyx damage (213). Syndecan-1 levels measured in circulation swere associated with severity of sepsis, acute kidney injury, need for intubation, and mortality (214–216). Endocan, a soluble dermatan sulfate proteoglycan in endothelial cells, can also be measured in circulation (217). Levels of endocan correlated with severity of sepsis and mortality (218) as well as cancer (219, 220).

Total sulphated glycosaminoglycans can also be measured in the urine based on the reaction of 1,9-dimethylmethylene blue with sulphated glycosaminoglycans. Measurement of specific constituents e.g., heparan sulfate can also be measured by degrading heparan sulfate with nitrous acid prior. Other glycosaminoglycans e.g., chondroitin and dermatan sulfate will not be degraded by nitrous acid. Urinary heparan sulfate would then be the difference between sulphated glycosaminoglycans with and without nitrous acid treatment. LC/MS allows for quantification of specific glycosaminoglycans as well, including dermatan sulfate, heparan sulfate, and keratan sulfate (206).

Components of tight junctions can also be measured in the plasma and damage to endothelial barrier function quantified. A well-written review by Vermette et al. (221) summarizes the studies on endothelial injury and tight junction proteins. For example, claudin-5 is expressed mainly in vascular endothelial cells and modulates the permeability of tight junctions (222). Levels of claudin-5 in serum have been demonstrated to correlate with severe plasma leakage in dengue infection (223). On the other hand, levels of vascular endothelial cadherin, also known as cadherin-5, a main component of adherens junction, did not demonstrate any association with endothelial permeability (223) although *in vitro* study demonstrated loss of vascular endothelial cadherin resulted in increased permeability (224). Other tight junction proteins such as zonula occludens 1 (ZO-1), can also be measured in human plasma, and was found to be associated with inflammation and cancer (225, 226). ZO-1 however, is also expressed in other cells and may not reflect endothelial injury alone.

Circulating endothelial cells (CECs) were first described in peripheral blood films (227) and higher levels of CECs were then observed in people with cardiovascular risk factors and acute myocardial infarction (228). CECs originate from the mature endothelium and can be considered as markers of endothelial injury and thus function (229). The phenotype and morphology of CECs varies widely depending on the underlying disease (230–232). Identification and differentiation of CECs from other cells namely endothelial progenitor cells (EPCs) is also challenging. EPCs are believed to originate from the bone marrow and are incorporated into new blood vessels at sites of ischemia and may function as part of an endogenous repair mechanism, rather than endothelial function itself (233–235). Improved characterization of CECs have allowed for immunomagnetic isolation and fluorescence activated cell sorting (FACS) to be used in identification of CECs (236) but further studies are needed to confirm that these methods are reproducible and accurate (237).

Endothelial microvesicles are small membranous particles released from endothelial cells and can be identified and measured from plasma through an array of methods including flow cytometry, ELISA, fluorescence confocal microscopy and electron microscopy (238). Clinical correlates with different types of endothelial dysfunction (238) and different triggers including infections, cancers and autoimmune conditions with endothelial microvesicles have been described (239). Further studies are also needed to better delineate the role of endothelial microvesicles in pathogenesis and thus its role in diagnostics and prognostication and hopefully therapeutics.

## Conclusion

Despite the wide array of techniques possible, our understanding of the human endothelium system is still not fully complete. The phenotypic heterogeneity of the endothelium cautions against extrapolating the physiology of endothelium in one organ bed to another organ bed. Invasive methods may still have to be utilized in organ beds that are not easily accessed or visualized and is likely not feasible in humans. Likewise, biomarkers measured in plasma may not reflect the local tissue environment and while microdialysis can measure the local tissue environment, it is still considered invasive and limited to accessible organs such as the skin. Specific limitations for each broad technique is described further in Table 1.

For the assessment of pre-clinical disease, the ideal technique should be non-invasive, accurate, reproducible, low-cost, and easily perform (240). Several techniques described above still require further evaluation to optimize and standardized protocols to ensure reproducibility, while some may benefit from a meta-analysis to ensure that measurements correlate with clinical end points. We are also in need of a prospective trial comparing the various techniques quantifying endothelial dysfunction and eventual clinical endpoints. It is unlikely that one technique is superior than the rest, but perhaps a combination of techniques will allow for a comprehensive evaluation of endothelial function with our current technological capabilities.

## Author Contributions

PC and TY conceptualized the review. PC drafted the manuscript and table which is original. PC, AT, and TY revised the manuscript critically for important intellectual content. All authors contributed to the article and approved the submitted version.

## Conflict of Interest

The authors declare that the research was conducted in the absence of any commercial or financial relationships that could be construed as a potential conflict of interest.

## Abbreviations

AFM: Atomic force microscopy
Ang: Angiopoietin
ANGPTL: Angiopoietin like proteins
AT: Antithrombin
CEC: Circulating endothelial cells
CFC: Capillary filtration coefficient
DAF-2: 4,5-diaminofluorescein
EDD: Endothelium dependent dilation
ezFMD: enclosed zone flow mediated dilation
ELISA: Enzyme linked immosorbent assay
FITC: Fluorescein isothiocyanate
Flk-1: Fetal liver kinase-1 (also known as VEGFR-2)
Flt-1: Fms-like tyrosine kinase-1 (also known as VEGFR-1)
FMD: Flow mediated dilation
FXa: Factor Xa
ICAM-1: Intercellular adhesion molecule-1
IDF: Incident dark field
LC/MS: Liquid chromatography/tandem mass spectrometry
LDF: Laser Doppler flowmetry
LDI: Laser Doppler imaging
LDPI: Laser Doppler perfusion imaging
L-FMC: Low-flow-mediated-constriction
MRI: Magnetic resonance imaging
NO: Nitric Oxide
OPS: Orthogonal polarization spectral
PECAM-1: Platelet endothelial cell adhesion molecule-1
PDGF: Platelet derived growth factor
PORH: Post-occlusive reactive hyperaemia
RHI: Reactive hyperaemia index
ROS: Reactive oxygen species
SDF: Sidestream dark field
TEER: Transendothelial electrical resistance
tPA: Tissue plasminogen activator
VCAM-1: Vascular cell adhesion molecule-1
VEGF: Vascular endothelial growth factor
VEGFR: Vascular endothelial growth factor receptor
ZO-1: Zonula occludens-1.

## References

[1] Jaffe EA. Cell biology of endothelial cells. Hum Pathol. (1987) 18:234–9. 10.1016/S0046-8177(87)80005-9 3546072

[2] Luft JH. Fine structures of capillary and endocapillary layer as revealed by ruthenium red. Fed Proc. (1966) 25:1773–83. 5927412

[3] Pries AR Secomb TW Gaehtgens P. The endothelial surface layer. Pflugers Arch. (2000) 440:653–66. 10.1007/s004240000307 11007304

[4] Bennett HS Luft JH Hampton JC. Morphological classifications of vertebrate blood capillaries. Am J Physiol. (1959) 196:381–90. 10.1152/ajplegacy.1959.196.2.381 13627187

[5] Kumar S West DC Ager A. Heterogeneity in endothelial cells from large vessels and microvessels. Differentiation. (1987) 36:57–70. 10.1111/j.1432-0436.1987.tb00181.x 2451631

[6] Aird WC. Phenotypic heterogeneity of the endothelium: I. Structure, function, and mechanisms. Circ Res. (2007) 100:158–73. 10.1161/01.RES.0000255691.76142.4a 17272818

[7] Okada H Takemura G Suzuki K Oda K Takada C Hotta Y. Three-dimensional ultrastructure of capillary endothelial glycocalyx under normal and experimental endotoxemic conditions. Crit Care. (2017) 21:261. 10.1186/s13054-017-1841-8 29058634 PMC5651619

[8] Florey. The endothelial cell. Br Med J. (1966) 2:487–90. 10.1136/bmj.2.5512.487 5913081 PMC1943522

[9] Ashford TP Frieiman DG. The role of the endothelium in the initial phases of thrombosis. an electron microscopic study. Am J Pathol. (1967) 50:257–73. 6016506 PMC1965234

[10] Furchgott RF Zawadzki JV. The obligatory role of endothelial cells in the relaxation of arterial smooth muscle by acetylcholine. Nature. (1980) 288:373–6. 10.1038/288373a0 6253831

[11] Catravas JD Lazo JS Dobuler KJ Mills LR Gillis CN. Pulmonary endothelial dysfunction in the presence or absence of interstitial injury induced by intratracheally injected bleomycin in rabbits. Am Rev Respir Dis. (1983) 128:740–6. 6194726 10.1164/arrd.1983.128.4.740

[12] Goligorsky MS. Endothelial cell dysfunction: can't live with it, how to live without it. Am J Physiol Renal Physiol. (2005) 288:F871–80. 10.1152/ajprenal.00333.2004 15821252

[13] Feletou M Vanhoutte PM. Endothelial dysfunction: a multifaceted disorder (The Wiggers Award Lecture). Am J Physiol Heart Circ Physiol. (2006) 291:H985–1002. 10.1152/ajpheart.00292.2006 16632549

[14] Reitsma S Slaaf DW Vink H Van Zandvoort MA Oude Egbrink MG. The endothelial glycocalyx: composition, functions, and visualization. Pflugers Arch. (2007) 454:345–59. 10.1007/s00424-007-0212-8 17256154 PMC1915585

[15] Simons M Alitalo K Annex BH Augustin HG Beam C Berk BC. State-of-the-art methods for evaluation of angiogenesis and tissue vascularization: a scientific statement from the American heart association. Circ Res. (2015) 116:e99–132. 10.1161/RES.0000000000000054 25931450 PMC8077227

[16] Nowak-Sliwinska P Alitalo K Allen E Anisimov A Aplin AC Auerbach R. Consensus guidelines for the use and interpretation of angiogenesis assays. Angiogenesis. (2018) 21:425–532. 10.1007/s10456-018-9613-x 29766399 PMC6237663

[17] Thijssen DH Black MA Pyke KE Padilla J Atkinson G Harris RA. Assessment of flow-mediated dilation in humans: a methodological and physiological guideline. Am J Physiol Heart Circ Physiol. (2011) 300:H2–12. 10.1152/ajpheart.00471.2010 20952670 PMC3023245

[18] Eskurza I Seals DR Desouza CA Tanaka H. Pharmacologic versus flow-mediated assessments of peripheral vascular endothelial vasodilatory function in humans. Am J Cardiol. (2001) 88:1067–9. 10.1016/S0002-9149(01)01997-X 11704016

[19] Weintraub WS Lüscher TF Pocock S. The perils of surrogate endpoints. Eur Heart J. (2015) 36:2212–8. 10.1093/eurheartj/ehv164 25975658 PMC4554958

[20] Newby David E Wright Robert A Labinjoh C Ludlam Christopher A Fox Keith AA Boon Nicholas A. Endothelial dysfunction, impaired endogenous fibrinolysis, and cigarette smoking. Circulation. (1999) 99:1411–5. 10.1161/01.CIR.99.11.1411 10086962

[21] Williams SB Goldfine AB Timimi FK Ting HH Roddy MA Simonson DC. Acute hyperglycemia attenuates endothelium-dependent vasodilation in humans *in vivo*. Circulation. (1998) 97:1695–701. 10.1161/01.CIR.97.17.1695 9591763

[22] Celermajer DS Sorensen KE Gooch VM Spiegelhalter DJ Miller OI Sullivan ID. Non-invasive detection of endothelial dysfunction in children and adults at risk of atherosclerosis. Lancet. (1992) 340:1111–5. 10.1016/0140-6736(92)93147-F 1359209

[23] Schachinger V Britten MB Zeiher AM. Prognostic impact of coronary vasodilator dysfunction on adverse long-term outcome of coronary heart disease. Circulation. (2000) 101:1899–906. 10.1161/01.CIR.101.16.1899 10779454

[24] Fischer D Landmesser U Spiekermann S Hilfiker-Kleiner D Hospely M Muller M. Cytochrome P450 2C9 is involved in flow-dependent vasodilation of peripheral conduit arteries in healthy subjects and in patients with chronic heart failure. Eur J Heart Fail. (2007) 9:770–5. 10.1016/j.ejheart.2007.05.005 17572144

[25] Hirano H Takama R Matsumoto R Tanaka H Hirano H Soh Z. Assessment of lower-limb vascular endothelial function based on enclosed zone flow-mediated dilation. Sci Rep. (2018) 8:9263. 10.1038/s41598-018-27392-3 29915185 PMC6006353

[26] Sandeman DD Pym CA Green EM Seamark C Shore AC Tooke JE. Microvascular vasodilatation in feet of newly diagnosed non-insulin dependent diabetic patients. BMJ. (1991) 302:1122–3. 10.1136/bmj.302.6785.1122 2043783 PMC1669799

[27] Debbabi H Bonnin P Ducluzeau PH Leftheriotis G Levy BI. Noninvasive assessment of endothelial function in the skin microcirculation. Am J Hypertens. (2010) 23:541–6. 10.1038/ajh.2010.10 20168305

[28] Walther G Obert P Dutheil F Chapier R Lesourd B Naughton G. Metabolic syndrome individuals with and without type 2 diabetes mellitus present generalized vascular dysfunction: cross-sectional study. Arterioscler Thromb Vasc Biol. (2015) 35:1022–9. 10.1161/ATVBAHA.114.304591 25657309

[29] Bonetti PO Pumper GM Higano ST Holmes DR Jr Kuvin JT Lerman A. Noninvasive identification of patients with early coronary atherosclerosis by assessment of digital reactive hyperemia. J Am Coll Cardiol. (2004) 44:2137–41. 10.1016/j.jacc.2004.08.062 15582310

[30] Davis JS Yeo TW Thomas JH Mcmillan M Darcy CJ Mcneil YR. Sepsis-associated microvascular dysfunction measured by peripheral arterial tonometry: an observational study. Crit Care. (2009) 13:R155. 10.1186/cc8055 19778457 PMC2784378

[31] Dharmashankar K Welsh A Wang J Kizhakekuttu TJ Ying R Gutterman DD. Nitric oxide synthase-dependent vasodilation of human subcutaneous arterioles correlates with noninvasive measurements of endothelial function. Am J Hypertens. (2012) 25:528–34. 10.1038/ajh.2012.8 22337207 PMC3328603

[32] Dirajlal-Fargo S Sattar A Kulkarni M Bowman E Funderburg N Mccomsey GA. HIV-positive youth who are perinatally infected have impaired endothelial function. AIDS. (2017) 31:1917–24. 10.1097/QAD.0000000000001556 28590332 PMC5578888

[33] Erre GL Piga M Fedele AL Mura S Piras A Cadoni ML. Prevalence and determinants of peripheral microvascular endothelial dysfunction in rheumatoid arthritis patients: a multicenter cross-sectional study. Mediators Inflamm. (2018) 2018:6548715. 10.1155/2018/6548715 29483841 PMC5816852

[34] Tooke JE. Microvascular haemodynamics in diabetes mellitus. Clin Sci. (1986) 70:119–25. 10.1042/cs0700119 3514078

[35] Heitzer T Schlinzig T Krohn K Meinertz T Munzel T. Endothelial dysfunction, oxidative stress, and risk of cardiovascular events in patients with coronary artery disease. Circulation. (2001) 104:2673–8. 10.1161/hc4601.099485 11723017

[36] Rosfors S Blomgren L. Venous occlusion plethysmography in patients with post-thrombotic venous claudication. J Vasc Surg. (2013) 58:722–6. 10.1016/j.jvs.2013.02.017 23548174

[37] Giannattasio C Mancia G. Arterial distensibility in humans. modulating mechanisms, alterations in diseases and effects of treatment. J Hypertens. (2002) 20:1889–99. 10.1097/00004872-200210000-00001 12359960

[38] Tomiyama H Yamashina A. Non-invasive vascular function tests: their pathophysiological background and clinical application. Circ J. (2010) 74:24–33. 10.1253/circj.CJ-09-0534 19920359

[39] Arrebola-Moreno AL Laclaustra M Kaski JC. Noninvasive assessment of endothelial function in clinical practice. Rev Esp Cardiol. (2012) 65:80–90. 10.1016/j.rec.2011.10.004 22099430

[40] Kohn JC Lampi MC Reinhart-King CA. Age-related vascular stiffening: causes and consequences. Front Genet. (2015) 6:112. 10.3389/fgene.2015.00112 25926844 PMC4396535

[41] Deng X Xiong F Li X Xiang B Li Z Wu X. Application of atomic force microscopy in cancer research. J Nanobiotechnol. (2018) 16:102. 10.1186/s12951-018-0428-0 30538002 PMC6288943

[42] Kiio TM Park S. Nano-scientific application of atomic force microscopy in pathology: from molecules to tissues. Int J Med Sci. (2020) 17:844–58. 10.7150/ijms.41805 32308537 PMC7163363

[43] Angus JA Cocks TM Mcpherson GA Broughton A. The acetylcholine paradox: a constrictor of human small coronary arteries even in the presence of endothelium. Clin Exp Pharmacol Physiol. (1991) 18:33–6. 10.1111/j.1440-1681.1991.tb01373.x 2032388

[44] Kichuk MR Seyedi N Zhang X Marboe CC Michler RE Addonizio LJ. Regulation of nitric oxide production in human coronary microvessels and the contribution of local kinin formation. Circulation. (1996) 94:44–51. 10.1161/01.CIR.94.1.44 8964116

[45] Myers J Irvine R Gillham J Macleod M Mires G Taggart M. Altered endothelial function in isolated human myometrial vessels induced by plasma from women with pre-eclampsia is not reproducible in isolated mouse vessels. Clin Sci. (2005) 108:457–62. 10.1042/CS20040343 15673282

[46] Fadini GP Avogaro A. Cell-based methods for *ex vivo* evaluation of human endothelial biology. Cardiovasc Res. (2010) 87:12–21. 10.1093/cvr/cvq119 20427336

[47] Onat D Brillon D Colombo PC Schmidt AM Human vascular endothelial cells: a model system for studying vascular inflammation in diabetes and atherosclerosis. Curr Diabetes Rep. (2011) 11:193–202. 10.1007/s11892-011-0182-2 PMC3311155 21337131

[48] Dababneh L Cikach F Alkukhun L Dweik RA Tonelli AR. Sublingual microcirculation in pulmonary arterial hypertension. Ann Am Thorac Soc. (2014) 11:504–12. 10.1513/AnnalsATS.201308-277OC 24601682 PMC4225801

[49] Jaarsma C Vink H Van Haare J Bekkers S Van Rooijen BD Backes WH. Non-invasive assessment of microvascular dysfunction in patients with microvascular angina. Int J Cardiol. (2017) 248:433–9. 10.1016/j.ijcard.2017.05.010 28733074

[50] Dilken O Ergin B Ince C. Assessment of sublingual microcirculation in critically ill patients: consensus and debate. Ann Transl Med. (2020) 8:793. 10.21037/atm.2020.03.222 32647718 PMC7333125

[51] Klaus S Heringlake M Bahlmann L. Bench-to-bedside review: microdialysis in intensive care medicine. Crit Care. (2004) 8:363–8. 10.1186/cc2882 15469599 PMC1065008

[52] Srinivasan B Kolli AR Esch MB Abaci HE Shuler ML Hickman JJ. TEER measurement techniques for *in vitro* barrier model systems. J Lab Autom. (2015) 20:107–26. 10.1177/2211068214561025 25586998 PMC4652793

[53] Mahajan SD Parikh NU Woodruff TM Jarvis JN Lopez M Hennon T. C5a alters blood-brain barrier integrity in a human *in vitro* model of systemic lupus erythematosus. Immunology. (2015) 146:130–43. 10.1111/imm.12489 26059553 PMC4552508

[54] Coronado-Velázquez D Betanzos A Serrano-Luna J Shibayama M. An *In vitro* model of the blood-brain barrier: naegleria fowleri affects the tight junction proteins and activates the microvascular endothelial cells. J Eukaryot Microbiol. (2018) 65:804–19. 10.1111/jeu.12522 29655298

[55] Yang X Meegan JE Jannaway M Coleman DC Yuan SY. A disintegrin and metalloproteinase 15-mediated glycocalyx shedding contributes to vascular leakage during inflammation. Cardiovasc Res. (2018) 114:1752–63. 10.1093/cvr/cvy167 29939250 PMC6198742

[56] Walsh SW. Plasma from preeclamptic women stimulates transendothelial migration of neutrophils. Reprod Sci. (2009) 16:320–5. 10.1177/1933719108327594 19087976 PMC2823372

[57] Schenning KJ Anderson S Alkayed NJ Hutchens MP. Hyperglycemia abolishes the protective effect of ischemic preconditioning in glomerular endothelial cells *in vitro*. Physiol Rep. (2015) 3:e12346. 10.14814/phy2.12346 25804266 PMC4393174

[58] Noonan J Grassia G Macritchie N Garside P Guzik TJ Bradshaw AC. A novel triple-cell two-dimensional model to study immune-vascular interplay in atherosclerosis. Front Immunol. (2019) 10:849. 10.3389/fimmu.2019.00849 31068936 PMC6491724

[59] Newby DE Mcleod AL Uren NG Flint L Ludlam CA Webb DJ. Impaired coronary tissue plasminogen activator release is associated with coronary atherosclerosis and cigarette smoking. Circulation. (2001) 103:1936–41. 10.1161/01.CIR.103.15.1936 11306520

[60] Oliver JJ Webb DJ Newby DE. Stimulated tissue plasminogen activator release as a marker of endothelial function in humans. Arterioscler Thromb Vasc Biol. (2005) 25:2470–9. 10.1161/01.ATV.0000189309.05924.88 16210566

[61] Dimitrievska S Gui L Weyers A Lin T Cai C Wu W. New functional tools for antithrombogenic activity assessment of live surface glycocalyx. Arterioscler Thromb Vasc Biol. (2016) 36:1847–53. 10.1161/ATVBAHA.116.308023 27386939 PMC5283952

[62] Yang YH Hwang KK Fitzgerald J Grossman JM Taylor M Hahn BH. Antibodies against the activated coagulation Factor X (FXa) in the antiphospholipid syndrome that interfere with the FXa inactivation by antithrombin. J Immunol. (2006) 77:8219. 10.4049/jimmunol.177.11.8219 17114499 PMC1950736

[63] Xing K Murthy S Liles WC Singh JM. Clinical utility of biomarkers of endothelial activation in sepsis–a systematic review. Crit Care. (2012) 16:R7. 10.1186/cc11145 22248019 PMC3396237

[64] Ricciuto DR Dos Santos CC Hawkes M Toltl LJ Conroy AL Rajwans N. Angiopoietin-1 and angiopoietin-2 as clinically informative prognostic biomarkers of morbidity and mortality in severe sepsis. Crit Care Med. (2011) 39:702–10. 10.1097/CCM.0b013e318206d285 21242795

[65] Eastwood KA Hunter AJ Patterson CC Mc Cance DR Young IS Holmes VA. The role of biomarkers in predicting pre-eclampsia in high-risk women. Ann Clin Biochem. (2020) 57:128–37. 10.1177/0004563219894022 31757167

[66] Tucker-Burden C Chappa P Krishnamoorthy M Gerwe BA Scharer CD Heimburg-Molinaro J. Lectins identify glycan biomarkers on glioblastoma-derived cancer stem cells. Stem Cells Dev. (2012) 21:2374–86. 10.1089/scd.2011.0369 22435486 PMC3425159

[67] Vlachopoulos C Xaplanteris P Aboyans V Brodmann M Cifkova R Cosentino F. The role of vascular biomarkers for primary and secondary prevention. a position paper from the European Society of Cardiology Working Group on peripheral circulation: endorsed by the Association for Research into Arterial Structure and Physiology (ARTERY) society. Atherosclerosis. (2015) 241:507–32. 10.1016/j.atherosclerosis.2015.05.007 26117398

[68] Ungvari Z Tarantini S Kiss T Wren JD Giles CB Griffin CT. Endothelial dysfunction and angiogenesis impairment in the ageing vasculature. Nat Rev Cardiol. (2018) 15:555–65. 10.1038/s41569-018-0030-z 29795441 PMC6612360

[69] Furchgott RF. Endothelium-derived relaxing factor: discovery, early studies, and identifcation as nitric oxide (Nobel Lecture). Angew. Chem. Int. Edn. (1999) 38:1870–80. 10.1002/(SICI)1521-3773(19990712)38:13/14<1870::AID-ANIE1870>3.0.CO;2-8 34182659

[70] Ignarro LJ Kadowitz PJ. The pharmacological and physiological role of cyclic GMP in vascular smooth muscle relaxation. Annu Rev Pharmacol Toxicol. (1985) 25:171–91. 10.1146/annurev.pa.25.040185.001131 2988418

[71] Vanhoutte PM Shimokawa H Feletou M Tang EH. Endothelial dysfunction and vascular disease - a 30th anniversary update. Acta Physiol. (2017) 219:22–96. 10.1111/apha.12646 26706498

[72] LuscherTFBartonM. Biology of the endothelium. Clin Cardiol. (1997) 20:II-3–10. 9422846

[73] Cines DB Pollak ES Buck CA Loscalzo J Zimmerman GA Mcever RP. Endothelial cells in physiology and in the pathophysiology of vascular disorders. Blood. (1998) 91:3527–61. 9572988

[74] Bonetti PO Lerman LO Lerman A. Endothelial dysfunction: a marker of atherosclerotic risk. Arterioscler Thromb Vasc Biol. (2003) 23:168–75. 10.1161/01.ATV.0000051384.43104.FC 12588755

[75] Yeboah J Crouse JR Hsu FC Burke GL Herrington DM. Brachial flow-mediated dilation predicts incident cardiovascular events in older adults: the cardiovascular health study. Circulation. (2007) 115:2390–7. 10.1161/CIRCULATIONAHA.106.678276 17452608

[76] Yeboah J Folsom AR Burke GL Johnson C Polak JF Post W. Predictive value of brachial flow-mediated dilation for incident cardiovascular events in a population-based study: the multi-ethnic study of atherosclerosis. Circulation. (2009) 120:502–9. 10.1161/CIRCULATIONAHA.109.864801 19635967 PMC2740975

[77] Leng GC Fowkes FG Donnan PT Housley E. Reactive hyperemia test in a random sample of the general population. J Vasc Surg. (1993) 17:479–86. 10.1016/0741-5214(93)90147-E 8445742

[78] Hudlická O. Effect of training on macro-and microcirculatory changes in exercise. Exerc Sport Sci Rev. (1977) 5:181–230. 10.1249/00003677-197700050-00007 357158

[79] Wilkinson IB Webb DJ. Venous occlusion plethysmography in cardiovascular research: methodology and clinical applications. Br J Clin Pharmacol. (2001) 52:631–46. 10.1046/j.0306-5251.2001.01495.x 11736874 PMC2014568

[80] Weber T Auer J O'rourke MF Kvas E Lassnig E Berent R. Arterial stiffness, wave reflections, and the risk of coronary artery disease. Circulation. (2004) 109:184–9. 10.1161/01.CIR.0000105767.94169.E3 14662706

[81] Lind L. Impact of ageing on the measurement of endothelium-dependent vasodilation. Pharmacol Rep. (2006) 58:41–6. 17332670

[82] Higashi Y Yoshizumi M. New methods to evaluate endothelial function: method for assessing endothelial function in humans using a strain-gauge plethysmography: nitric oxide-dependent and -independent vasodilation. J Pharmacol Sci. (2003) 93:399–404. 10.1254/jphs.93.399 14737008

[83] Silva AM Schaan BD Signori LU Plentz RD Moreno H Jr Bertoluci MC. Microalbuminuria is associated with impaired arterial and venous endothelium-dependent vasodilation in patients with Type 2 diabetes. J Endocrinol Invest. (2010) 33:696–700. 10.1007/BF03346672 20354354

[84] Aellig WH. A new technique for recording compliance of human hand veins. Br J Clin Pharmacol. (1981) 11:237–43. 10.1111/j.1365-2125.1981.tb00527.x 7213525 PMC1401623

[85] Gutterman DD Chabowski DS Kadlec AO Durand MJ Freed JK Ait-Aissa K. The human microcirculation: regulation of flow and beyond. Circ Res. (2016) 118:157–72. 10.1161/CIRCRESAHA.115.305364 26837746 PMC4742348

[86] Corretti MC Anderson TJ Benjamin EJ Celermajer D Charbonneau F Creager MA. Guidelines for the ultrasound assessment of endothelial-dependent flow-mediated vasodilation of the brachial artery: a report of the International Brachial Artery Reactivity Task Force. J Am Coll Cardiol. (2002) 39:257–65. 10.1016/S0735-1097(01)01746-6 11788217

[87] Mitchell GF Parise H Vita JA Larson MG Warner E Keaney JF Jr. Local shear stress and brachial artery flow-mediated dilation: the Framingham heart study. Hypertension. (2004) 44:134–9. 10.1161/01.HYP.0000137305.77635.68 15249547

[88] Bonjorno Junior JC Caruso FR Mendes RG Da Silva TR Biazon TMPDC Rangel F. Noninvasive measurements of hemodynamic, autonomic and endothelial function as predictors of mortality in sepsis: a prospective cohort study. PLoS ONE. (2019) 14:e0213239. 10.1371/journal.pone.0213239 30856206 PMC6411260

[89] Perregaux D Chaudhuri A Rao S Airen A Wilson M Sung BH. Brachial vascular reactivity in blacks. Hypertension. (2000) 36:866–71. 10.1161/01.HYP.36.5.866 11082158

[90] Takase B Goto T Hamabe A Uehata A Kuroda K Satomura K. Flow-mediated dilation in brachial artery in the second half of pregnancy and prediction of pre-eclampsia. J Hum Hypertension. (2003) 17:697–704. 10.1038/sj.jhh.1001599 14504628

[91] Hoskins PR Fish PJ Mcdicken WN Moran C. Developments in cardiovascular ultrasound. part 2: arterial applications. Med Biol Eng Comput. (1998) 36:259–69. 10.1007/BF02522469 9747563

[92] Bots ML Westerink J Rabelink TJ De Koning EJ. Assessment of flow-mediated vasodilatation (FMD) of the brachial artery: effects of technical aspects of the FMD measurement on the FMD response. Eur Heart J. (2004) 26:363–8. 10.1093/eurheartj/ehi017 15618057

[93] Pyke KE Tschakovsky ME. The relationship between shear stress and flow-mediated dilatation: implications for the assessment of endothelial function. J Physiol. (2005) 568:357–69. 10.1113/jphysiol.2005.089755 16051630 PMC1474741

[94] Leeson CP Robinson M Francis JM Robson MD Channon KM Neubauer S. Cardiovascular magnetic resonance imaging for non-invasive assessment of vascular function: validation against ultrasound. J Cardiovasc Magn Reson. (2006) 8:381–7. 10.1080/10976640500526993 16669182

[95] Gori T Dragoni S Lisi M Di Stolfo G Sonnati S Fineschi M. Conduit artery constriction mediated by low flow a novel noninvasive method for the assessment of vascular function. J Am Coll Cardiol. (2008) 51:1953–8. 10.1016/j.jacc.2008.01.049 18482663

[96] Gori T Grotti S Dragoni S Lisi M Di Stolfo G Sonnati S. Assessment of vascular function: flow-mediated constriction complements the information of flow-mediated dilatation. Heart. (2010) 96:141–7. 10.1136/hrt.2009.167213 19858140

[97] Kuvin JT Patel AR Sliney KA Pandian NG Sheffy J Schnall RP. Assessment of peripheral vascular endothelial function with finger arterial pulse wave amplitude. Am Heart J. (2003) 146:168–74. 10.1016/S0002-8703(03)00094-2 12851627

[98] Axtell AL Gomari FA Cooke JP. Assessing endothelial vasodilator function with the Endo-PAT 2000. J Vis Exp. (2010) 2167. 10.3791/2167 20972417 PMC3143035

[99] Matsuzawa Y Kwon TG Lennon RJ Lerman LO Lerman A. Prognostic value of flow-mediated vasodilation in brachial artery and fingertip artery for cardiovascular events: a systematic review and meta-analysis. J Am Heart Assoc. (2015) 4:e002270. 10.1161/JAHA.115.002270 26567372 PMC4845238

[100] Hewlett A vZJ. The rate of blood flow in the arm. Heart. (1909) 1:631–46.

[101] Lewis T Grant R Observations upon reactive hyperaemia in man. Heart. (1925) 12:120.

[102] Whitney R. The measurement of volume changes in human limbs. J Physiol. (1953) 121:1–27. 10.1113/jphysiol.1953.sp004926 13085295 PMC1366051

[103] Greenfield AD Whitney RJ Mowbray JF. Methods for the investigation of peripheral blood flow. Br Med Bull. (1963) 19:101–9. 10.1093/oxfordjournals.bmb.a070026 13950177

[104] Schraibman I Mott D Naylor G Charlesworth D. Impedance plethysmography: evaluation of a simplified system of electrodes for the measurement of blood flow in the lower limb. Br J Surg. (1976) 63:413–6. 10.1002/bjs.1800630520 1268485

[105] Junejo RT Ray CJ Marshall JM. Cuff inflation time significantly affects blood flow recorded with venous occlusion plethysmography. Eur J Appl Physiol. (2019) 119:665–74. 10.1007/s00421-018-04056-8 30617468 PMC6394686

[106] Roustit M Cracowski JL. Non-invasive assessment of skin microvascular function in humans: an insight into methods. Microcirculation. (2012) 19:47–64. 10.1111/j.1549-8719.2011.00129.x 21883640

[107] Stern MD. *In vivo* evaluation of microcirculation by coherent light scattering. Nature. (1975) 254:56–8. 10.1038/254056a0 1113878

[108] Roustit M Blaise S Millet C Cracowski JL. Reproducibility and methodological issues of skin post-occlusive and thermal hyperemia assessed by single-point laser Doppler flowmetry. Microvasc Res. (2010) 79:102–8. 10.1016/j.mvr.2010.01.001 20064535

[109] Cracowski JL Minson CT Salvat-Melis M Halliwill JR. Methodological issues in the assessment of skin microvascular endothelial function in humans. Trends Pharmacol Sci. (2006) 27:503–8. 10.1016/j.tips.2006.07.008 16876881

[110] Millet C Roustit M Blaise S Cracowski JL. Comparison between laser speckle contrast imaging and laser Doppler imaging to assess skin blood flow in humans. Microvasc Res. (2011) 82:147–51. 10.1016/j.mvr.2011.06.006 21745482

[111] Zötterman J Mirdell R Horsten S Farnebo S Tesselaar E. Methodological concerns with laser speckle contrast imaging in clinical evaluation of microcirculation. PLoS ONE. (2017) 12:e0174703. 10.1371/journal.pone.0174703 28358906 PMC5373607

[112] Babos L Jarai Z Nemcsik J. Evaluation of microvascular reactivity with laser Doppler flowmetry in chronic kidney disease. World J Nephrol. (2013) 2:77–83. 10.5527/wjn.v2.i3.77 24255889 PMC3832914

[113] Greaney JL Saunders EFH Santhanam L Alexander LM. Oxidative stress contributes to microvascular endothelial dysfunction in men and women with major depressive disorder. Circ Res. (2019) 124:564–74. 10.1161/CIRCRESAHA.118.313764 30582458 PMC6375800

[114] Heeman W Steenbergen W Van Dam G Boerma EC. Clinical applications of laser speckle contrast imaging: a review. J Biomed Opt. (2019) 24:1–11. 10.1117/1.JBO.24.8.080901 31385481 PMC6983474

[115] Jaffe EA Nachman RL Becker CG Minick CR. Culture of human endothelial cells derived from umbilical veins. identification by morphologic and immunologic criteria. J Clin Invest. (1973) 52:2745–56. 10.1172/JCI107470 4355998 PMC302542

[116] Aman J Weijers EM Van Nieuw Amerongen GP Malik AB Van Hinsbergh VWM. Using cultured endothelial cells to study endothelial barrier dysfunction: challenges and opportunities. Am J Physiol Lung Cell Mol Physiol. (2016) 311:L453–66. 10.1152/ajplung.00393.2015 27343194 PMC5504427

[117] Cai H Dikalov S Griendling KK Harrison DG. Detection of reactive oxygen species and nitric oxide in vascular cells and tissues: comparison of sensitivity and specificity. Methods Mol Med. (2007) 139:293–311. 10.1007/978-1-59745-571-8_20 18287681

[118] Leikert JF Rathel TR Muller C Vollmar AM Dirsch VM. Reliable *in vitro* measurement of nitric oxide released from endothelial cells using low concentrations of the fluorescent probe 4,5-diaminofluorescein. FEBS Lett. (2001) 506:131–4. 10.1016/S0014-5793(01)02901-5 11591386

[119] Bohm B Oberhoffer R. Vascular health determinants in children. Cardiovasc Diagn Ther. (2019) 9:S269–80. 10.21037/cdt.2018.09.16 31737535 PMC6837937

[120] Van Bortel LM Laurent S Boutouyrie P Chowienczyk P Cruickshank JK De Backer T. Expert consensus document on the measurement of aortic stiffness in daily practice using carotid-femoral pulse wave velocity. J Hypertens. (2012) 30:445–8. 10.1097/HJH.0b013e32834fa8b0 22278144

[121] Wassertheurer S Kropf J Weber T Van Der Giet M Baulmann J Ammer M. A new oscillometric method for pulse wave analysis: comparison with a common tonometric method. J Hum Hypertens. (2010) 24:498–504. 10.1038/jhh.2010.27 20237499 PMC2907506

[122] Vriz O Driussi C La Carrubba S Di Bello V Zito C Carerj S. Comparison of sequentially measured Aloka echo-tracking one-point pulse wave velocity with SphygmoCor carotid-femoral pulse wave velocity. SAGE Open Med. (2013) 1:2050312113507563. 10.1177/2050312113507563 26770685 PMC4687782

[123] Williams B Mancia G Spiering W Agabiti Rosei E Azizi M Burnier M. 2018 Practice Guidelines for the management of arterial hypertension of the European Society of Hypertension and the European Society of Cardiology: ESH/ESC task force for the management of arterial hypertension. J Hypertens. (2018) 36:2284–309. 10.1097/HJH.0000000000001961 30379783

[124] Shahin Y Khan JA Chetter I. Angiotensin converting enzyme inhibitors effect on arterial stiffness and wave reflections: a meta-analysis and meta-regression of randomised controlled trials. Atherosclerosis. (2012) 221:18–33. 10.1016/j.atherosclerosis.2011.12.005 22209214

[125] Guerin AP Blacher J Pannier B Marchais SJ Safar ME London GM. Impact of aortic stiffness attenuation on survival of patients in end-stage renal failure. Circulation. (2001) 103:987–92. 10.1161/01.CIR.103.7.987 11181474

[126] Mulvany MJ Halpern W. Contractile properties of small arterial resistance vessels in spontaneously hypertensive and normotensive rats. Circ Res. (1977) 41:19–26. 10.1161/01.RES.41.1.19 862138

[127] Duling BR Gore RW Dacey RG Jr Damon DN. Methods for isolation, cannulation, and *in vitro* study of single microvessels. Am J Physiol. (1981) 241:H108–16. 10.1152/ajpheart.1981.241.1.H108 7195654

[128] Schjorring OL Carlsson R Simonsen U. Pressure myography to study the function and structure of isolated small arteries. Methods Mol Biol. (2015) 1339:277–95. 10.1007/978-1-4939-2929-0_19 26445796

[129] Angulo J Vallejo S El Assar M Garcia-Septiem J Sanchez-Ferrer CF Rodriguez-Manas L. Age-related differences in the effects of alpha and gamma peroxisome proliferator-activated receptor subtype agonists on endothelial vasodilation in human microvessels. Exp Gerontol. (2012) 47:734–40. 10.1016/j.exger.2012.06.014 22776133

[130] Ives SJ Park SY Kwon OS Gifford JR Andtbacka RHI Hyngstrom JR. TRPV(1) channels in human skeletal muscle feed arteries: implications for vascular function. Exp Physiol. (2017) 102:1245–58. 10.1113/EP086223 28681979 PMC5578896

[131] Ford TJ Rocchiccioli P Good R Mcentegart M Eteiba H Watkins S. Systemic microvascular dysfunction in microvascular and vasospastic angina. Eur Heart J. (2018) 39:4086–97. 10.1093/eurheartj/ehy529 30165438 PMC6284165

[132] Binnig G Quate CF Gerber C. Atomic force microscope. Phys Rev Lett. (1986) 56:930–3. 10.1103/PhysRevLett.56.930 10033323

[133] Oberleithner H Peters W Kusche-Vihrog K Korte S Schillers H Kliche K. Salt overload damages the glycocalyx sodium barrier of vascular endothelium. Pflugers Arch. (2011) 462:519–28. 10.1007/s00424-011-0999-1 21796337 PMC3170475

[134] Wiesinger A Peters W Chappell D Kentrup D Reuter S Pavenstädt H. Nanomechanics of the endothelial glycocalyx in experimental sepsis. PLoS ONE. (2013) 8:e80905. 10.1371/journal.pone.0080905 24278345 PMC3835794

[135] Ince C Boerma EC Cecconi M De Backer D Shapiro NI Duranteau J. Second consensus on the assessment of sublingual microcirculation in critically ill patients: results from a task force of the European Society of Intensive Care Medicine. Intensive Care Med. (2018) 44:281–99. 10.1007/s00134-018-5070-7 29411044

[136] Ince C. The microcirculation is the motor of sepsis. Crit Care. (2005) 9(Suppl. 4):S13–9. 10.1186/cc3753 16168069 PMC3226164

[137] Groner W Winkelman JW Harris AG Ince C Bouma GJ Messmer K. Orthogonal polarization spectral imaging: a new method for study of the microcirculation. Nat Med. (1999) 5:1209–12. 10.1038/13529 10502828

[138] Goedhart PT Khalilzada M Bezemer R Merza J Ince C. Sidestream Dark Field (SDF) imaging: a novel stroboscopic LED ring-based imaging modality for clinical assessment of the microcirculation. Opt Express. (2007) 15:15101–14. 10.1364/OE.15.015101 19550794

[139] Aykut G Veenstra G Scorcella C Ince C Boerma C. Cytocam-IDF (incident dark field illumination) imaging for bedside monitoring of the microcirculation. Intensive Care Med Exp. (2015) 3:40. 10.1186/s40635-015-0040-7 26215807 PMC4512989

[140] Massey MJ Shapiro NI. A guide to human *in vivo* microcirculatory flow image analysis. Crit Care. (2016) 20:35. 10.1186/s13054-016-1213-9 26861691 PMC4748457

[141] Lee DH Dane MJ van den Berg BM Boels MG van Teeffelen JW de Mutsert R. NEO study group. Deeper penetration of erythrocytes into the endothelial glycocalyx is associated with impaired microvascular perfusion. PLoS One. (2014) 9:e96477. 10.1371/journal.pone.0096477 24816787 PMC4015985

[142] Edul VS Enrico C Laviolle B Vazquez AR Ince C Dubin A. Quantitative assessment of the microcirculation in healthy volunteers and in patients with septic shock. Crit Care Med. (2012) 40:1443–8. 10.1097/CCM.0b013e31823dae59 22430243

[143] De Backer D Donadello K Sakr Y Ospina-Tascon G Salgado D Scolletta S. Microcirculatory alterations in patients with severe sepsis: impact of time of assessment and relationship with outcome. Crit Care Med. (2013) 41:791–9. 10.1097/CCM.0b013e3182742e8b 23318492

[144] Vellinga NA Boerma EC Koopmans M Donati A Dubin A Shapiro NI. International study on microcirculatory shock occurrence in acutely ill patients. Crit Care Med. (2015) 43:48–56. 10.1097/CCM.0000000000000553 25126880

[145] Bito L Davson H Levin EE Murray M Snider N. The concentrations of free amino acids and other electrolytes in cerebrospinal fluid, *in vivo* dialysate of brain, and blood plasma of the dog. J Neurochem. (1966) 13:1057–67. 10.1111/j.1471-4159.1966.tb04265.x 5924657

[146] Friston D Laycock H Nagy I Want EJ. Microdialysis workflow for metabotyping superficial pathologies: application to burn injury. Analy Chem. (2019) 91:6541–8. 10.1021/acs.analchem.8b05615 31021084 PMC6533596

[147] Kaul S Williams TD Lunte CE Faiman MD. LC-MS/MS determination of carbamathione in microdialysis samples from rat brain and plasma. J Pharm Biomed Anal. (2010) 51:186–91. 10.1016/j.jpba.2009.07.026 19709836 PMC2760994

[148] La Favor JD Dubis GS Yan H White JD Nelson MA Anderson EJ. Microvascular endothelial dysfunction in sedentary, obese humans is mediated by NADPH oxidase: influence of exercise training. Arterioscler Thromb Vasc Biol. (2016) 36:2412–20. 10.1161/ATVBAHA.116.308339 27765769 PMC5123754

[149] Dejana E. Endothelial cell-cell junctions: happy together. Nat Rev Mol Cell Biol. (2004) 5:261–70. 10.1038/nrm1357 15071551

[150] Sukriti S Tauseef M Yazbeck P Mehta D. Mechanisms regulating endothelial permeability. Pulm Circ. (2014) 4:535–51. 10.1086/677356 25610592 PMC4278616

[151] Gamble J Gartside IB Christ F. A reassessment of mercury in silastic strain gauge plethysmography for microvascular permeability assessment in man. J Physiol. (1993) 464:407–22. 10.1113/jphysiol.1993.sp019642 8229810 PMC1175393

[152] Roztocil K Prerovsky I Studnicka J Oliva I Halova J. Capillary filtration during postischaemic hyperaemia in human limbs. Physiol Bohemoslov. (1978) 27:31–5. 148055

[153] Ando S Imaizumi T Harada S Hirooka Y Takeshita A. Atrial natriuretic peptide increases human capillary filtration and venous distensibility. J Hypertens. (1992) 10:451–7. 10.1097/00004872-199205000-00008 1317906

[154] Jaap AJ Shore AC Gartside IB Gamble J Tooke JE. Increased microvascular fluid permeability in young type 1 (insulin-dependent) diabetic patients. Diabetologia. (1993) 36:648–52. 10.1007/BF00404075 8359583

[155] Mahy IR Lewis DM Tooke JE. Limb capillary filtration coefficient in human subjects: the importance of the site of measurement. Physiol Meas. (1998) 19:339–43. 10.1088/0967-3334/19/3/002 9735884

[156] Bauer A Bruegger D Gamble J Christ F. Influence of different cuff inflation protocols on capillary filtration capacity in human calves – a congestion plethysmography study. J Physiol. (2002) 543:1025–31. 10.1113/jphysiol.2002.018291 12231656 PMC2290538

[157] Crone C Olesen SP. Electrical resistance of brain microvascular endothelium. Brain Res. (1982) 241:49–55. 10.1016/0006-8993(82)91227-6 6980688

[158] Robinson BD Shaji CA Lomas A Tharakan B. Measurement of microvascular endothelial barrier dysfunction and hyperpermeability *in vitro*. Methods Mol Biol. (2018) 1717:237–42. 10.1007/978-1-4939-7526-6_19 29468597

[159] Williams MC Wissig SL. The permeability of muscle capillaries to horseradish peroxidase. J Cell Biol. (1975) 66:531–55. 10.1083/jcb.66.3.531 169269 PMC2109454

[160] Duffy SL Murphy JT. Colorimetric assay to quantify macromolecule diffusion across endothelial monolayers. Biotechniques. (2001) 31:495–6, 498, 500–1. 10.2144/01313st02 11570492

[161] Simionescu M Gafencu A Antohe F. Transcytosis of plasma macromolecules in endothelial cells: a cell biological survey. Microsc Res Tech. (2002) 57:269–88. 10.1002/jemt.10086 12112439

[162] Ghim M Alpresa P Yang SW Braakman ST Gray SG Sherwin SJ. Visualization of three pathways for macromolecule transport across cultured endothelium and their modification by flow. Am J Physiol Heart Circ Physiol. (2017) 313:H959–73. 10.1152/ajpheart.00218.2017 28754719 PMC5792200

[163] Uhlig S Yang Y Waade J Wittenberg C Babendreyer A Kuebler WM. Differential regulation of lung endothelial permeability *in vitro* and *in situ*. Cell Physiol Biochem. (2014) 34:1–19. 10.1159/000362980 24977477

[164] Potter DR Damiano ER. The hydrodynamically relevant endothelial cell glycocalyx observed *in vivo* is absent *in vitro*. Circ Res. (2008) 102:770–6. 10.1161/CIRCRESAHA.107.160226 18258858

[165] De Mesy Bentley Karen L. An 11-μm-thick glycocalyx? Arterioscler Thromb Vasc Biol. (2011) 31:1712–3. 10.1161/ATVBAHA.111.229849 21775768

[166] Ebong EE Macaluso FP Spray DC Tarbell JM. Imaging the endothelial glycocalyx *in vitro* by rapid freezing/freeze substitution transmission electron microscopy. Arterioscler Thromb Vasc Biol. (2011) 31:1908–15. 10.1161/ATVBAHA.111.225268 21474821 PMC3141106

[167] Buchanan CF Verbridge SS Vlachos PP Rylander MN. Flow shear stress regulates endothelial barrier function and expression of angiogenic factors in a 3D microfluidic tumor vascular model. Cell Adh Migr. (2014) 8:517–24. 10.4161/19336918.2014.970001 25482628 PMC4594487

[168] Kang H Cancel LM Tarbell JM. Effect of shear stress on water and LDL transport through cultured endothelial cell monolayers. Atherosclerosis. (2014) 233:682–90. 10.1016/j.atherosclerosis.2014.01.056 24583416 PMC3979984

[169] Henderson-Toth CE Jahnsen ED Jamarani R Al-Roubaie S Jones EA. The glycocalyx is present as soon as blood flow is initiated and is required for normal vascular development. Dev Biol. (2012) 369:330–9. 10.1016/j.ydbio.2012.07.009 22820069

[170] Hesh CA Qiu Y Lam WA. Vascularized microfluidics and the blood-endothelium interface. Micromachines (Basel). (2019) 11:18. 10.3390/mi11010018 31878018 PMC7019435

[171] Musafargani S Mishra S Gulyas M Mahalakshmi P Archunan G Padmanabhan P. Blood brain barrier: a tissue engineered microfluidic chip. J Neurosci Methods. (2020) 331:108525. 10.1016/j.jneumeth.2019.108525 31756396

[172] Destefano JG Jamieson JJ Linville RM Searson PC. Benchmarking *in vitro* tissue-engineered blood-brain barrier models. Fluids Barriers CNS. (2018) 15:32. 10.1186/s12987-018-0117-2 30514389 PMC6280508

[173] Matute-Bello G Downey G Moore BB Groshong SD Matthay MA Slutsky AS. An official American thoracic society workshop report: features and measurements of experimental acute lung injury in animals. Am J Respir Cell Mol Biol. (2011) 44:725–38. 10.1165/rcmb.2009-0210ST 21531958 PMC7328339

[174] Feng L Stern DM Pile-Spellman J. Human endothelium: endovascular biopsy and molecular analysis. Radiology. (1999) 212:655–64. 10.1148/radiology.212.3.r99au28655 10478228

[175] Colombo PC Ashton AW Celaj S Talreja A Banchs JE Dubois NB. Biopsy coupled to quantitative immunofluorescence: a new method to study the human vascular endothelium. J Appl Physiol. (2002) 92:1331–8. 10.1152/japplphysiol.00680.2001 11842075

[176] Shelton JL Wang L Cepinskas G Sandig M Inculet R Mccormack DG. Albumin leak across human pulmonary microvascular vs. umbilical vein endothelial cells under septic conditions. Microvasc Res. (2006) 71:40–7. 10.1016/j.mvr.2005.11.003 16376951

[177] Feng L Stern DM Pile-Spellman J. Human endothelium: endovascular biopsy and molecular analysis. Radiology. (1999) 212:655–64. 10.1148/radiology.212.3.r99au28655 10478228

[178] Otto M Bittinger F Kriegsmann J Kirkpatrick CJ. Differential adhesion of polymorphous neutrophilic granulocytes to macro- and microvascular endothelial cells under flow conditions. Pathobiology. (2001) 69:159–71. 10.1159/000048771 11872962

[179] Hekman CM Loskutoff DJ. Fibrinolytic pathways and the endothelium. Semin Thromb Hemost. (1987) 13:514–27. 10.1055/s-2007-1003527 2827312

[180] Newby DE Wright RA Ludlam CA Fox KA Boon NA Webb DJ. An *in vivo* model for the assessment of acute fibrinolytic capacity of the endothelium. Thromb Haemost. (1997) 78:1242–8. 10.1055/s-0038-1657722 9364992

[181] Hunter AL Shah ASV Langrish JP Raftis JB Lucking AJ Brittan M. Fire simulation and cardiovascular health in firefighters. Circulation. (2017) 135:1284–95. 10.1161/CIRCULATIONAHA.116.025711 28373523 PMC5377985

[182] Noh RM Venkatasubramanian S Daga S Langrish J Mills NL Lang NN. Cardiometabolic effects of a novel SIRT1 activator, SRT2104, in people with type 2 diabetes mellitus. Open Heart. (2017) 4:e000647. 10.1136/openhrt-2017-000647 28912956 PMC5588958

[183] Smith DT Hoetzer GL Greiner JJ Stauffer BL Desouza CA. Effects of ageing and regular aerobic exercise on endothelial fibrinolytic capacity in humans. J Physiol. (2003) 546:289–98. 10.1113/jphysiol.2002.027870 12509496 PMC2342457

[184] Paulus P Jennewein C Zacharowski K. Biomarkers of endothelial dysfunction: can they help us deciphering systemic inflammation and sepsis? Biomarkers. (2011) 16(Suppl. 1):S11–21. 10.3109/1354750X.2011.587893 21707440

[185] Skibsted S Jones AE Puskarich MA Arnold R Sherwin R Trzeciak S. Biomarkers of endothelial cell activation in early sepsis. Shock. (2013) 39:427–32. 10.1097/SHK.0b013e3182903f0d 23524845 PMC3670087

[186] Mankhambo LA Banda DL Jeffers G White SA Balmer P Nkhoma S. The role of angiogenic factors in predicting clinical outcome in severe bacterial infection in Malawian children. Crit Care. (2010) 14:R91. 10.1186/cc9025 20492647 PMC2911728

[187] Palud A Parmentier-Decrucq E Pastre J De Freitas Caires N Lassalle P Mathieu D. Evaluation of endothelial biomarkers as predictors of organ failures in septic shock patients. Cytokine. (2015) 73:213–8. 10.1016/j.cyto.2015.02.013 25794660

[188] Santulli G. Angiopoietin-like proteins: a comprehensive look. Front Endocrinol. (2014) 5:4. 10.3389/fendo.2014.00004 24478758 PMC3899539

[189] Doi Y Ninomiya T Hirakawa Y Takahashi O Mukai N Hata J. Angiopoietin-like protein 2 and risk of type 2 diabetes in a general Japanese population: the Hisayama study. Diabetes Care. (2013) 36:98–100. 10.2337/dc12-0166 22966088 PMC3526200

[190] Tuuri AL Jauhiainen MS Ehnholm CP Tikkanen MJ Nicholls MG Kaaja RJ. Elevated serum angiopoietin-like protein 6 in women with subsequent pregnancy-induced hypertension: a preliminary study. Hypertens Pregnancy. (2013) 32:203–13. 10.3109/10641955.2013.784783 23905605

[191] Kansas GS. Selectins and their ligands: current concepts and controversies. Blood. (1996) 88:3259–87. 10.1182/blood.V88.9.3259.bloodjournal8893259 8896391

[192] Patel KD Cuvelier SL Wiehler S. Selectins: critical mediators of leukocyte recruitment. Semin Immunol. (2002) 14:73–81. 10.1006/smim.2001.0344 11978079

[193] Lertkiatmongkol P Liao D Mei H Hu Y Newman PJ. Endothelial functions of platelet/endothelial cell adhesion molecule-1 (CD31). Curr Opin Hematol. (2016) 23:253–9. 10.1097/MOH.0000000000000239 27055047 PMC4986701

[194] Dvorak HF Nagy JA Feng D Brown LF Dvorak AM. Vascular permeability factor/vascular endothelial growth factor and the significance of microvascular hyperpermeability in angiogenesis. Curr Top Microbiol Immunol. (1999) 237:97–132. 10.1007/978-3-642-59953-8_6 9893348

[195] Van Der Flier M Van Leeuwen HJ Van Kessel KP Kimpen JL Hoepelman AI Geelen SP. Plasma vascular endothelial growth factor in severe sepsis. Shock. (2005) 23:35–8. 10.1097/01.shk.0000150728.91155.41 15614129

[196] Greco M Palumbo C Sicuro F Lobreglio G. Soluble Fms-like tyrosine kinase-1 is a marker of endothelial dysfunction during sepsis. J Clin Med Res. (2018) 10:700–6. 10.14740/jocmr3505w 30116440 PMC6089578

[197] Tangren JS Thadhani R. Novel preeclampsia diagnostics and real-world applications. Hypertension. (2019) 74:740–2. 10.1161/HYPERTENSIONAHA.119.12908 31401879

[198] Park DJ Seo AN Yoon C Ku GY Coit DG Strong VE. Serum VEGF-A and tumor vessel VEGFR-2 levels predict survival in caucasian but not asian patients undergoing resection for gastric adenocarcinoma. Ann Surg Oncol. (2015) 22(Suppl. 3):S1508–15. 10.1245/s10434-015-4790-y 26259755 PMC4872865

[199] Sawada M Oishi T Komatsu H Sato S Chikumi J Nonaka M. Serum vascular endothelial growth factor A and vascular endothelial growth factor receptor 2 as prognostic biomarkers for uterine cervical cancer. Int J Clin Oncol. (2019) 24:1612–9. 10.1007/s10147-019-01495-x 31236742

[200] Andrae J Gallini R Betsholtz C. Role of platelet-derived growth factors in physiology and medicine. Genes Dev. (2008) 22:1276–312. 10.1101/gad.1653708 18483217 PMC2732412

[201] Alvarez RH Kantarjian HM Cortes JE. Biology of platelet-derived growth factor and its involvement in disease. Mayo Clin Proc. (2006) 81:1241–57. 10.4065/81.9.1241 16970222

[202] Blazquez-Medela AM Garcia-Ortiz L Gomez-Marcos MA Recio-Rodriguez JI Sanchez-Rodriguez A Lopez-Novoa JM. Increased plasma soluble endoglin levels as an indicator of cardiovascular alterations in hypertensive and diabetic patients. BMC Med. (2010) 8:86. 10.1186/1741-7015-8-86 21171985 PMC3012013

[203] Saita E Miura K Suzuki-Sugihara N Miyata K Ikemura N Ohmori R. Plasma soluble endoglin levels are inversely associated with the severity of coronary atherosclerosis—brief report. Arterioscler Thromb Vasc Biol. (2017) 37:49–52. 10.1161/ATVBAHA.116.308494 27789477

[204] Lepedda AJ De Muro P Capobianco G Formato M. Significance of urinary glycosaminoglycans/proteoglycans in the evaluation of type 1 and type 2 diabetes complications. J Diabetes Complications. (2017) 31:149–55. 10.1016/j.jdiacomp.2016.10.013 27842978

[205] Jura-Póltorak A Olczyk P Chalas-Lipka A Komosinska-Vassev K Kuznik-Trocha K Winsz-Szczotka K. Urinary sulphated glycosaminoglycans excretion in obese patients with type 2 diabetes mellitus treated with metformin. Arch Physiol Biochem. (2019) [Epub ahead of print]. 10.1080/13813455.2019.1697889 31815550

[206] Lin HY Lee CL Lo YT Tu RY Chang YH Chang CY. An at-risk population screening program for mucopolysaccharidoses by measuring urinary glycosaminoglycans in Taiwan. Diagnostics. (2019) 9:140. 10.3390/diagnostics9040140 31590383 PMC6963841

[207] Schmidt EP Overdier KH Sun X Lin L Liu X Yang Y. Urinary glycosaminoglycans predict outcomes in septic shock and acute respiratory distress syndrome. Am J Respir Crit Care Med. (2016) 194:439–49. 10.1164/rccm.201511-2281OC 26926297 PMC5003330

[208] Tang TH Alonso S Ng LF Thein TL Pang VJ Leo YS. Increased serum hyaluronic acid and heparan sulfate in dengue fever: association with plasma leakage and disease severity. Sci Rep. (2017) 7:46191. 10.1038/srep46191 28393899 PMC5385535

[209] Inkinen N Pettilä V Lakkisto P Kuitunen A Jukarainen S Bendel S. Association of endothelial and glycocalyx injury biomarkers with fluid administration, development of acute kidney injury, and 90-day mortality: data from the FINNAKI observational study. Ann Intensive Care. (2019) 9:103. 10.1186/s13613-019-0575-y 31512003 PMC6738365

[210] Yeo TW Weinberg JB Lampah DA Kenangalem E Bush P Chen Y. Glycocalyx breakdown is associated with severe disease and fatal outcome in plasmodium falciparum malaria. Clin Infect Dis. (2019) 69:1712–20. 10.1093/cid/ciz038 30753363 PMC6821254

[211] Uchimido R Schmidt EP Shapiro NI. The glycocalyx: a novel diagnostic and therapeutic target in sepsis. Crit Care. (2019) 23:16. 10.1186/s13054-018-2292-6 30654825 PMC6337861

[212] Saunders S Jalkanen M O'farrell S Bernfield M. Molecular cloning of syndecan, an integral membrane proteoglycan. J Cell Biol. (1989) 108:1547–56. 10.1083/jcb.108.4.1547 2494194 PMC2115498

[213] Teng YH-F Aquino RS Park PW. Molecular functions of syndecan-1 in disease. Matrix Biol. (2012) 31:3–16. 10.1016/j.matbio.2011.10.001 22033227 PMC3568394

[214] Sallisalmi M Tenhunen J Yang R Oksala N Pettilä V. Vascular adhesion protein-1 and syndecan-1 in septic shock. Acta Anaesthesiol Scand. (2012) 56:316–22. 10.1111/j.1399-6576.2011.02578.x 22150439

[215] Neves FMDO Meneses GC Sousa NEA Menezes RRPPBD Parahyba MC Martins AMC. Syndecan-1 in acute decompensated heart failure–association with renal function and mortality. Circul J. (2015) 79:1511–9. 10.1253/circj.CJ-14-1195 25891890

[216] Puskarich MA Cornelius DC Tharp J Nandi U Jones AE. Plasma syndecan-1 levels identify a cohort of patients with severe sepsis at high risk for intubation after large-volume intravenous fluid resuscitation. J Crit Care. (2016) 36:125–9. 10.1016/j.jcrc.2016.06.027 27546760 PMC6371967

[217] Bechard D Gentina T Delehedde M Scherpereel A Lyon M Aumercier M. Endocan is a novel chondroitin sulfate/dermatan sulfate proteoglycan that promotes hepatocyte growth factor/scatter factor mitogenic activity. J Biol Chem. (2001) 276:48341–9. 10.1074/jbc.M108395200 11590178

[218] Scherpereel A Depontieu F Grigoriu B Cavestri B Tsicopoulos A Gentina T. Endocan, a new endothelial marker in human sepsis. Crit Care Med. (2006) 34:532–7. 10.1097/01.CCM.0000198525.82124.74 16424738

[219] Scherpereel A Gentina T Grigoriu B Senechal S Janin A Tsicopoulos A. Overexpression of endocan induces tumor formation. Cancer Res. (2003) 63:6084–9. 14522939

[220] Grigoriu BD Depontieu F Scherpereel A Gourcerol D Devos P Ouatas T. Endocan expression and relationship with survival in human non-small cell lung cancer. Clin Cancer Res. (2006) 12:4575–82. 10.1158/1078-0432.CCR-06-0185 16899604

[221] Vermette D Hu P Canarie MF Funaro M Glover J Pierce RW. Tight junction structure, function, and assessment in the critically ill: a systematic review. Intensive Care Medi Exp. (2018) 6:37–37. 10.1186/s40635-018-0203-4 30259344 PMC6158145

[222] Günzel D Yu ASL. Claudins and the modulation of tight junction permeability. Physiol Rev. (2013) 93:525–69. 10.1152/physrev.00019.2012 23589827 PMC3768107

[223] Suwarto S Sasmono RT Sinto R Ibrahim E Suryamin M. Association of endothelial glycocalyx and tight and adherens junctions with severity of plasma leakage in dengue infection. J Infect Dis. (2017) 215:992–9. 10.1093/infdis/jix041 28453844 PMC5407050

[224] Singh S Anupriya MG Modak A Sreekumar E. Dengue virus or NS1 protein induces trans-endothelial cell permeability associated with VE-Cadherin and RhoA phosphorylation in HMEC-1 cells preventable by angiopoietin-1. J Gen Virol. (2018) 99:1658–70. 10.1099/jgv.0.001163 30355397

[225] Karthikeyan A Mohan P Chinnakali P Vairappan B. Elevated systemic zonula occludens 1 is positively correlated with inflammation in cirrhosis. Clin Chim Acta. (2018) 480:193–8. 10.1016/j.cca.2018.02.017 29458051

[226] Ram AK Pottakat B Vairappan B. Increased systemic zonula occludens 1 associated with inflammation and independent biomarker in patients with hepatocellular carcinoma. BMC Cancer. (2018) 18:572. 10.1186/s12885-018-4484-5 29776350 PMC5960107

[227] Bouvier C Circulating endothelium as an indication of vascular injury. Thromb Diath Haemorrh. (1970) 40:163.

[228] Hladovec J Prerovský I Stanek V Fabián J. Circulating endothelial cells in acute myocardial infarction and angina pectoris. Klin Wochenschr. (1978) 56:1033–6. 10.1007/BF01476669 723184

[229] Gendron N Smadja DM. Circulating endothelial cells: a new biomarker of endothelial dysfunction in hematological diseases. Ann Biol Clin. (2016) 74:395–404. 10.1684/abc.2016.1160 27492692

[230] Dignat-George F Sampol J. Circulating endothelial cells in vascular disorders: new insights into an old concept. Eur J Haematol. (2000) 65:215–20. 10.1034/j.1600-0609.2000.065004215.x 11073162

[231] Woywodt A Streiber F De Groot K Regelsberger H Haller H Haubitz M. Circulating endothelial cells as markers for ANCA-associated small-vessel vasculitis. Lancet. (2003) 361:206–10. 10.1016/S0140-6736(03)12269-6 12547543

[232] Zhang K Yin F Lin L. Circulating endothelial cells and chronic kidney disease. Biomed Res Int. (2014) 2014:364738. 10.1155/2014/364738 24949439 PMC4052150

[233] Shi Q Rafii S Wu MH Wijelath ES Yu C Ishida A. Evidence for circulating bone marrow-derived endothelial cells. Blood. (1998) 92:362–7. 10.1182/blood.V92.2.362.414k38_362_367 9657732

[234] Hill JM Zalos G Halcox JP Schenke WH Waclawiw MA Quyyumi AA. Circulating endothelial progenitor cells, vascular function, and cardiovascular risk. N Engl J Med. (2003) 348:593–600. 10.1056/NEJMoa022287 12584367

[235] Urbich C Dimmeler S. Endothelial progenitor cells: characterization and role in vascular biology. Circ Res. (2004) 95:343–53. 10.1161/01.RES.0000137877.89448.78 15321944

[236] Erdbruegger U Haubitz M Woywodt A. Circulating endothelial cells: a novel marker of endothelial damage. Clin Chim Acta. (2006) 373:17–26. 10.1016/j.cca.2006.05.016 16836991

[237] Delorme B Basire A Gentile C Sabatier F Monsonis F Desouches C. Presence of endothelial progenitor cells, distinct from mature endothelial cells, within human CD146+ blood cells. Thromb Haemost. (2005) 94:1270–9. 10.1160/TH05-07-0499 16411405

[238] Liu ML Williams KJ. Microvesicles: potential markers and mediators of endothelial dysfunction. Curr Opin Endocrinol Diabetes Obes. (2012) 19:121–7. 10.1097/MED.0b013e32835057e9 22248645 PMC5031554

[239] Vítková V Živný J Janota J. Endothelial cell-derived microvesicles: potential mediators and biomarkers of pathologic processes. Biomark Med. (2018) 12:161–75. 10.2217/bmm-2017-0182 29327597

[240] Deanfield JE Halcox JP Rabelink TJ. Endothelial function and dysfunction: testing and clinical relevance. Circulation. (2007) 115:1285–95. 10.1161/CIRCULATIONAHA.106.652859 17353456

